# Structural determinants of the SINE B2 element embedded in the long non-coding RNA activator of translation *AS Uchl1*

**DOI:** 10.1038/s41598-017-14908-6

**Published:** 2018-02-16

**Authors:** Peter Podbevšek, Francesca Fasolo, Carlotta Bon, Laura Cimatti, Sabine Reißer, Piero Carninci, Giovanni Bussi, Silvia Zucchelli, Janez Plavec, Stefano Gustincich

**Affiliations:** 1 Area of Neuroscience, SISSA, Trieste, Italy; 2 Slovenian NMR Centre, National Institute of Chemistry, Ljubljana, Slovenia; 3 EN-FIST Centre of Excellence, Ljubljana, Slovenia; 4 Molecular and Statistical Biophysics, SISSA, Trieste, Italy; 5 Division of Genomic Technologies, RIKEN Center for Life Science Technologies, Yokohama, Japan; 6 Department of Health Sciences, Università del Piemonte Orientale, Novara, Italy; 7 Faculty of Chemistry and Chemical Technology, University of Ljubljana, Ljubljana, Slovenia; 8 Department of Neuroscience and Brain Technologies, Istituto Italiano di Tecnologia, Genova, Italy

## Abstract

Pervasive transcription of mammalian genomes leads to a previously underestimated level of complexity in gene regulatory networks. Recently, we have identified a new functional class of natural and synthetic antisense long non-coding RNAs (lncRNA) that increases translation of partially overlapping sense mRNAs. These molecules were named SINEUPs, as they require an embedded inverted SINE B2 element for their UP-regulation of translation. Mouse AS Uchl1 is the representative member of natural SINEUPs. It was originally discovered for its role in increasing translation of Uchl1 mRNA, a gene associated with neurodegenerative diseases. Here we present the secondary structure of the SINE B2 Transposable Element (TE) embedded in AS Uchl1. We find that specific structural regions, containing a short hairpin, are required for the ability of AS Uchl1 RNA to increase translation of its target mRNA. We also provide a high-resolution structure of the relevant hairpin, based on NMR observables. Our results highlight the importance of structural determinants in embedded TEs for their activity as functional domains in lncRNAs.

## Introduction

Transposable elements (TEs) are mobile repetitive sequences that represent about 50% of the mammalian genomes. Previously referred to as genomic “junk”, it is now increasingly acknowledged that TEs contribute to a wide range of biological processes, ultimately promoting genome evolution through rearrangements of genome structure and function. Specific TEs can be regulatory DNA elements acting as promoters or enhancers^1–3^ as well as platforms to recruit transcription factors and chromatin remodelling complexes^4–6^. They can be natural sources of regulatory sequences, co-opted to rewire gene regulatory networks^7^. In physiological conditions, TE mobilization has been implicated in the evolution of embryonic stem cells^8^ and placenta^9^, in pluripotency maintenance^10,11^, in the development of the mouse neocortex^12,13^ and in immune responses triggered by proinflammatory interferon-γ^7^. In addition, TEs can be “exonized” in protein-coding transcripts and function as regulatory RNA domains, contributing to alternative splicing^14^ or originating miRNAs^15,16^. Recently, a number of studies have highlighted a functional role of TEs when embedded in long non-coding RNAs (lncRNAs). lncRNAs are defined as transcripts >200 nt in length with no coding capability. lncRNAs have been implicated in a variety of biological functions, regulating gene expression at many levels^17,18^. Based on updated catalogues of annotated lncRNAs^19^, it is estimated that more than 80% of human and 66% of mouse lncRNAs contain at least one exonized TE. As a consequence, 40% of human and 33% of mouse lncRNA sequences derive from TEs^20–22^. Selective families of TEs can be found enriched or depleted in sub-classes of lncRNAs in the two species, arguing for an active role of TEs in evolution and function of lncRNAs^20,22,23^. In this context, exonized (or embedded) TEs would represent the molecular basis for domain organization in lncRNAs^24,25^. However, direct evidence for domain functionality of embedded TEs is still limited to few examples. Human Alu elements embedded in a group of lncRNAs, called 1/2-sbsRNAs, provide an RNA recognition motif that base pairs with complementary Alu sequences in the 3′ untranslated region of protein-coding mRNAs to drive their decay^26^. An embedded Alu element is involved in recruiting proteins of the Polycomb Repressor Complex, thus modulating the biological activity of ANRIL, a lncRNA associated to coronary artery disease^27^.

Recently, we have demonstrated that an embedded inverted SINE B2 (invSINEB2) element acts as a functional domain in antisense (AS) Uchl1, an AS lncRNA able to increase translation of partially-overlapping protein-coding sense Uchl1 mRNA^28^. The translation enhancer function of AS Uchl1 depends on two RNA domains. At the 5′, the region overlapping with Uchl1 mRNA confers target mRNA specificity and is referred to as the Binding Domain (BD). In the non-overlapping sequence, the invSINEB2 element provides the translation activation function of AS Uchl1 and it is defined as the Effector Domain (ED). By solely combing BD with the invSINEB2 ED it is possible to retain the gene-specific up-regulation function of AS Uchl1^25,28^. Similarly, AS Uchl1 activity can be transferred to a synthetic construct by changing the antisense sequence in the BD^25,29,30^. Bioinformatic mining of the FANTOM3 collection of lncRNAs identified at least 31 naturally occurring AS lncRNAs overlapping with protein-coding mRNAs at the 5′ end and containing an invSINEB2 element^28^. Altogether, AS Uchl1 can be considered the representative member of a new functional class of natural and synthetic AS lncRNAs named SINEUPs, as they require a SINE B2 element to UP-regulate translation^31^. While natural SINEUPs are investigated for their role in translational control under stress, synthetic SINEUPs are currently under intense scrutiny for their use in therapeutic intervention for gene-dosage dependent genetic diseases such as haploinsufficiences.

Despite recent advances in supporting the functional role of embedded TEs as lncRNA domains, it still remains to be determined how these elements can retain a specific biological function despite very poor sequence conservation. The crucial challenge is the understanding of the structure/function relationship between embedded TEs and lncRNAs and the definition of structural determinants that contribute to their biological function.

This work describes the structural features of the invSINEB2 element as ED embedded in AS Uchl1 RNA. Chemical footprinting combined with functional validation in murine neuroblastoma cells identified a short hairpin as key structural determinant for the ability of AS Uchl1 to increase translation. A high-resolution structure of the hairpin has been derived based on NMR observables.

Our results strengthen the role of structural determinants in embedded TEs for their activity as functional domains in lncRNAs.

## Results

### Secondary structure of the invSINEB2 TE embedded in AS Uchl1 RNA

A 183 nt construct (invSINEB2/183) corresponding to the invSINEB2 element of AS Uchl1 was *in vitro* transcribed from a plasmid and prepared for secondary structure determination using chemical footprinting. DMS (dimethyl sulfate) and CMCT (1-cyclohexyl-(2-morpholinoethyl)carbodiimide metho-p-toluene sulfonate) were used as methylating agents. DMS preferentially methylates positions N1 and N3 of adenines and cytosines, respectively, while CMCT methylates position N3 of uridines and to a lesser extent N1 of guanines. The level of methylation is directly related to the accessibility of potential modification sites to solvent. Therefore, hydrogen bonded nucleotides are not methylated, while non-hydrogen bonded are. The methylation sites were analysed by reverse transcribing RNA into cDNA starting from a fluorescently labelled DNA primer. The DNA oligos were analysed on large sequencing gels and visualized on a densitometer (Figure S1). Data from footprinting studies has been used as an input for restrained mFOLD secondary structure prediction^32^. It is noteworthy that data from either of the methylating agents was sufficient for an unambiguous secondary structure determination.

**Figure 1:**
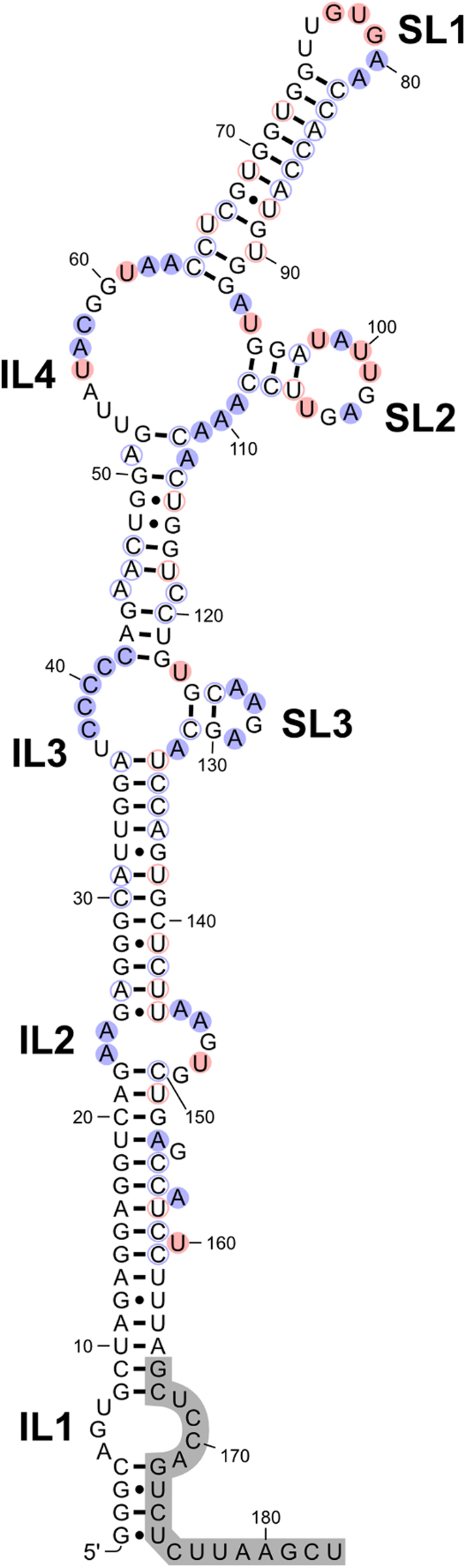
Secondary structure of the invSINEB2/183 effector domain of AS Uchl1. DMS and CMCT reactive nucleotides are shaded in blue and red, respectively. Internal loops and stem-loops are labelled as ILx and SLx, respectively. Non-reactive nucleotides are only circled. The segment shaded in grey corresponds to the DNA primer hybridization site.

The invSINEB2/183 RNA folds into a structure with mostly helical secondary structure elements (Fig. 1). It exhibits several bulges, asymmetric internal loops and hairpins. Consisting of nucleotides 5–7 and 168–171, the internal loop (IL1) could not be directly probed with DMS or CMCT due to hybridization of the fluorescent DNA primer to the 3′ of the RNA. This internal loop is followed by a helical region, which contains three single nucleotide bulges, with nucleotides G154, A157 and U160 showing weak reactivity with methylating agents. The asymmetric internal loop (IL2) is comprised of nucleotides 23–25 and 144–149. Data suggests that G25:U144 base pair is not formed according to chemical footprinting as U144 is reactive with CMCT, while U143 remains protected. This is to be expected, due to the relatively low stability of G:U base pairs, especially at the termini of helical regions. The invSINEB2/183 construct features two more internal loops, which branch out into short hairpins. Comprised of nucleotides 37–41 and 123–132, the internal loop (IL3) is branched into a short stem-tetraloop element (SL3). Similarly, nucleotides 53–63 and 93–111 form a larger internal loop (IL4) with a stem-octaloop motif (SL2). The terminal hairpin (SL1) includes nucleotides 64–92 and exhibits a G/C rich stem with an A/U rich loop region. All stem nucleotides up to C64 and G92 are protected from methylation, including U66:U90 mismatch nucleotides. On the other hand, loop nucleotides G77, U78 and G79 are all susceptible to methylation by CMCT. Importantly, A80 can be methylated by DMS while A81 exhibits very weak reactivity. Partial solvent access suggests that the two A:U base pairs are involved in an equilibrium between opened and closed states.

### The terminal SL1 hairpin contributes to AS Uchl1 ability to increase UchL1 protein levels

**Figure 2:**
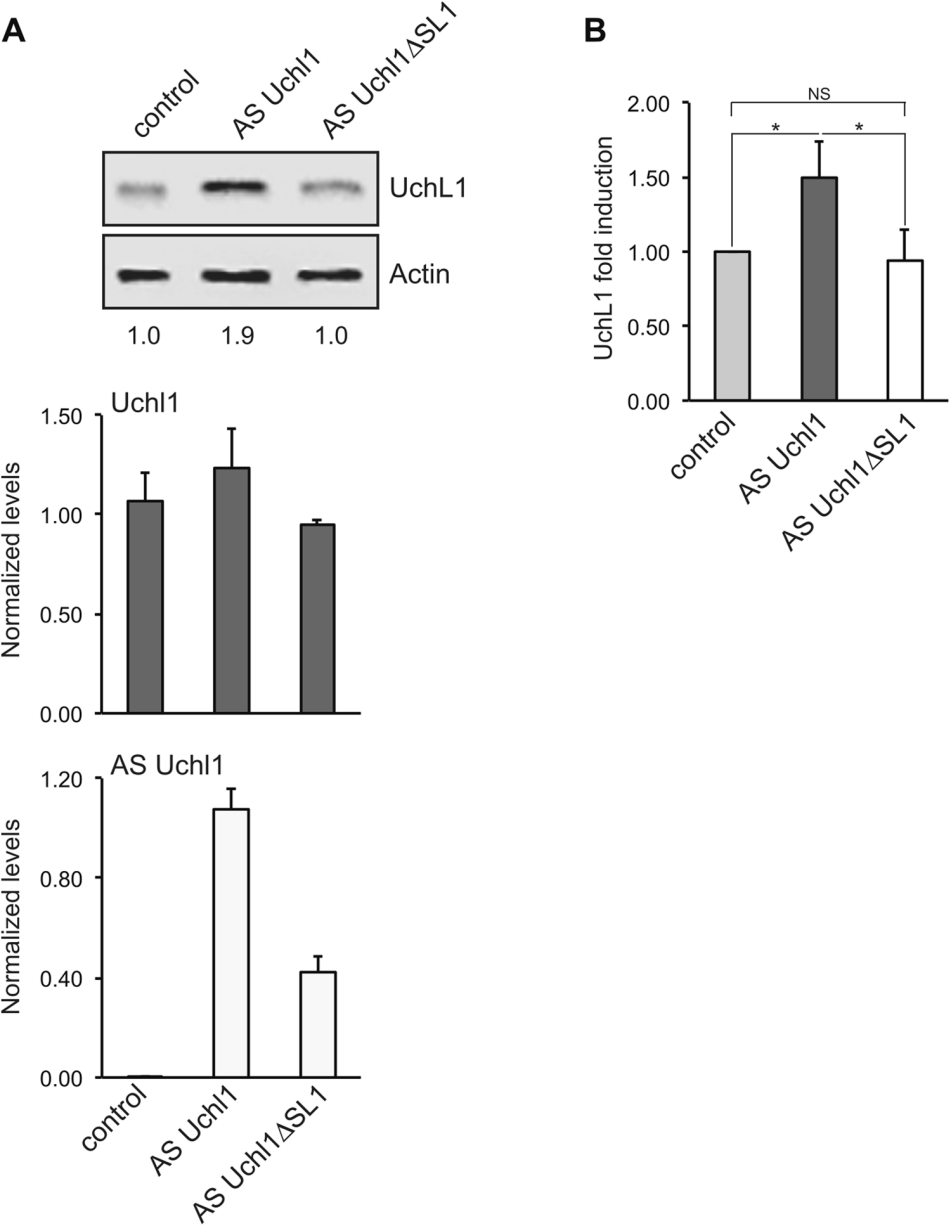
The SL1 hairpin contributes to AS Uchl1 activity. (**A**) Murine neuroblastoma N2a cells were transfected with AS Uchl1 and ΔSL1 mutant constructs. Control cells were transfected with an empty control plasmid. 48 hours after transfection, cells were lysed and processed for protein (top) and RNA (bottom) levels. Western blot was performed with anti-UCHL1 antibody. β-actin was used as loading control. Fold-induction was calculated on Western blot images normalized to β-actin and relative to empty control samples. Expressions of Uchl1 mRNA (gray bars) and AS Uchl1 (white bars) were monitored by qRT-PCR using specific primers. Data indicate mean ± st. dev. Data are representative of N = 5 independent replicas. (**B**) Graphical representation of AS Uchl1 and ΔSL1 translation enhancement activity on endogenous Uchl1 mRNA in N2a cells (N = 5). *p = 0.01; NS, not significant (p > 0.5).

We have previously shown that the embedded invSINEB2 element acts as ED in natural and synthetic SINEUPs^31^. Deletion of invSINEB2 sequence, but not of the embedded Alu repeat, abolishes UchL1 protein up-regulation mediated by AS Uchl1 in mouse neuroblastoma cell lines^28^. Here we investigated whether secondary structure components of invSINEB2 affect the function of AS Uchl1 RNA. We focused our attention on the terminal SL1 since chemical footprinting suggests it is a stable secondary structure element within invSINEB2. Furthermore, as terminal hairpin, it should be easily accessible to RNA-protein interactions. We disrupted the terminal hairpin structure by deleting nucleotides 68–77 of invSINEB2 (ΔSL1) from the full length AS Uchl1 (ΔSL1 mutant). According to mFOLD structure prediction, this deletion would not affect the stable helical regions, but rearrangements in IL4 are expected. To investigate invSINEB2-ΔSL1 activity when embedded in full length AS Uchl1, we took advantage of murine neuroblastoma Neuro2a cells, as they express Uchl1 mRNA but do not contain detectable levels of endogenous AS Uchl1. AS Uchl1 activity was defined as UchL1 protein increase in the presence of unchanged mRNA levels, as quantified by western blotting and qRT-PCR, respectively. AS Uchl1 caused an ~1.9-fold increase in UchL1 protein levels while maintaining stable Uchl1 mRNA levels, as expected for a post-transcriptional regulatory mechanism. Interestingly, the ΔSL1 deletion mutant abolished the ability of AS Uchl1 RNA to up-regulate UchL1 protein levels (Fig. 2A). Indeed, UchL1 amounts were comparable in cells transfected with ΔSL1 mutant and in control samples, while differing in a statistically significant manner from cells with full length AS Uchl1 (Fig. 2B).

Taken together, these data indicate that the terminal SL1 hairpin of embedded invSINEB2 is a structural determinant required for AS Uchl1 ability to increase protein levels as synthesized from its target mRNA.

### Solution state NMR reveals a triloop structure

**Figure 3:**
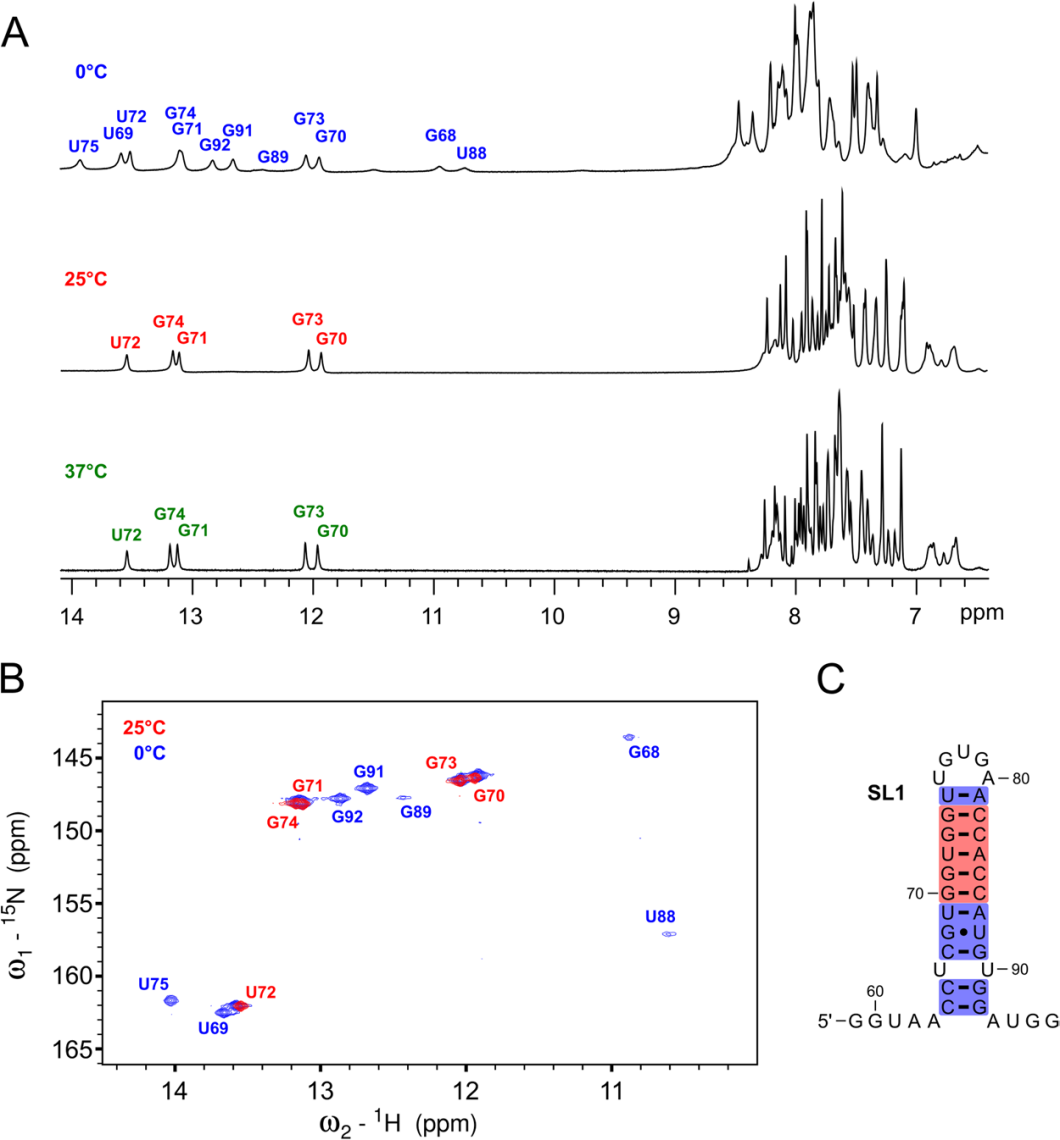
invSINEB2/38 adopts a single structure in solution. (**A**) Imino and aromatic regions of ^1^H NMR spectra of 38 nt RNA in 5% ^2^H_2_O/95% H_2_O acquired at 0, 25 and 37 °C. Imino ^1^H signals are assigned. (**B**) Imino region of the ^15^N HSQC NMR spectra. Signals observed at 25 and 0 °C are in red and blue, respectively. (**C**) Secondary structure of the invSINEB2/38 construct. Base pairs with imino signals observable at 25 and 0 °C are shaded in red and blue, respectively.

To further elucidate the structure of SL1 NMR spectroscopy in solution has been utilized. The full length invSINEB2/183 element and the ΔSL1 construct did not give resolved NMR spectra (Fig. S2) due to their high molecular weights (>55 kDa). Therefore, a 38 nt RNA fragment (invSINEB2/38) corresponding to nucleotides 59–96 of the invSINEB2/183 has been synthesized for NMR studies. Analysis of 1D ^1^H spectra revealed that a single species is formed in solution (Fig. 3A). Although experiments were carried out at 25 °C it is noteworthy that the structure is stable also at the physiological temperature of 37 °C. With the use of a natural abundance and a uniformly ^13^C, ^15^N-labeled RNA most imino, amino, aromatic and anomeric resonances could be assigned with a set of triple resonance NMR experiments. A sequential walk could be traced in NOESY spectra of the 38 nt construct (Figure S3) although some cross-peaks could not be directly observed due to signal overlap (Figure S4). Perusal of ^15^N HSQC NMR spectra (Fig. 3B) revealed that the secondary structure of the invSINEB2/38 RNA is in agreement with the structure of this fragment within the entire invSINEB2/183 RNA. The RNA folds into a stable hairpin structure, with five and four nucleotide overhangs on 5′ and 3′ ends, respectively (Fig. 3C).

The stem region with observable imino protons extends from nucleotides C64-U75 and A81-G92 and is comprised of seven G:C, three A:U and one G:U base pairs with an additional U:U mismatch. The ^15^N HSQC spectrum acquired at 25 °C contains the signals of imino protons G70-G74 comprising the core of the stem (Fig. 3B). Lowering the temperature to 0 °C revealed additional signals for base paired nucleotides, whose imino protons are to a lesser extent protected from exchange with solvent. These include the terminal U75:A81 base pair and the region from C64:G92 to U69:A87 including the G68:U88 base pair. Interestingly, no signal could be observed for U76 imino protons, which would indicate that U76:A80 base pair is dynamic or not formed at all. On the other hand, U75 exhibits a narrow imino proton resonance at 0 °C, suggesting that base pair U75:A81 is stable. Furthermore, imino proton signals of G68, U88 and G89 are broad even at 0 °C indicating that base pairs G68:U88 and C67:G89 are relatively weak, which is most likely due to the destabilizing effect of the adjacent U66:U90 mismatch for which no imino resonances can be observed.

2D COSY and TOCSY spectra exhibit no intense H1′/H2′ cross-peaks indicating that sugar puckers of all nucleotides are C3′-endo. Intensities of intra-nucleotide H6,H8/H1′ NOESY cross-peaks suggest that all nucleotides in the stem region are in *anti* conformation. However, due to inconclusive analysis of H6,H8/H1′ cross-peak intensities in the loop region, MD simulations did not include any χ angle restraints for nucleotides U75-A81.

**Figure 4:**
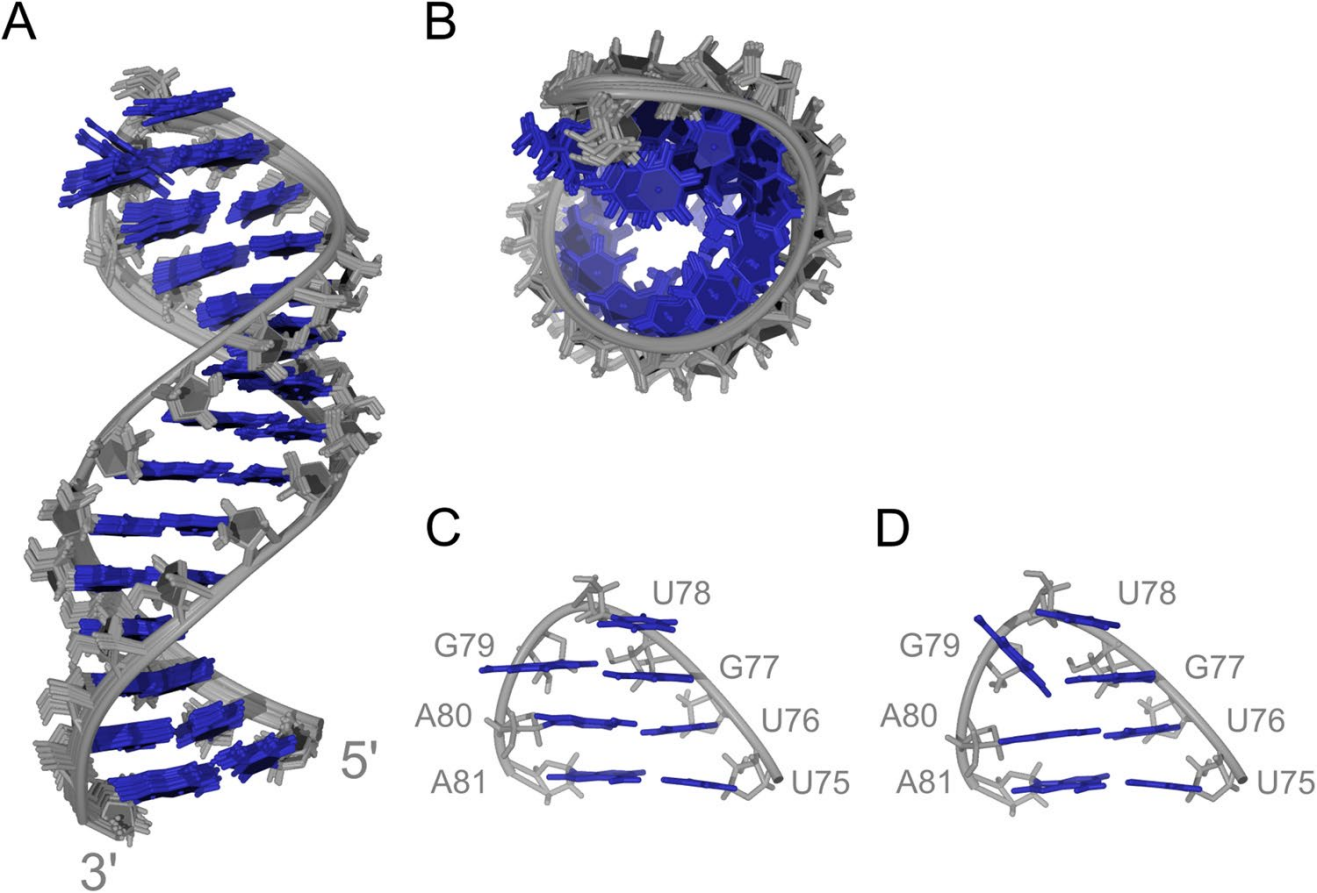
High-resolution structure of SL1. (**A**) Side and (**B**) top views of the 10 lowest energy structures of the 29 nt hairpin. (**C** and **D**) Representative structures of loops with orientations of G77, U78 and G79 bases.

A high resolution structure of the hairpin RNA without the 5′ and 3′ overhangs (29 nt) has been derived on the basis of NMR observables and restrained molecular dynamics simulations. A set of 100 structures was calculated using a simulated annealing protocol from which 10 of the lowest energy structures were selected and subjected to subsequent energy minimization. The hairpin adopts an A-type helical stem, which is terminated by a triloop (Fig. 4). The final set of 10 structures exhibits a pairwise heavy atom RMSD of 0.7 Å (Table 1). The stem region nucleotides (C64-U75 and A81-G92) give an average RMSD value of 0.5 Å, while the loop nucleotides (G77-G79) exhibit a higher average RMSD of 0.7 Å. The stem region exhibits average rise and twist parameters of 3.0 Å and 30.3 Å, respectively. In comparison, the rise and twist parameters in standard A-type RNA are 2.8 Å and 32.8 Å, respectively. Due to the lack of observable imino proton signals for U76, hydrogen bond restraints for the U76:A80 base pair were not included in the calculations. In addition, all backbone dihedral angle restraints were omitted for the loop and two adjacent A:U base pair nucleotides (U75-A81).

**Table 1:** NMR and refinement statistics.

	**5LSN**
**NMR distance and dihedral constraints**	
Distance restraints	
Total NOE	125
Intra-residue	34
Inter-residue	91
Sequential (|*i* – *j*| = 1)	77
Nonsequential (|*i* – *j*| > 1)	14
Hydrogen bonds	29
Total dihedral angle restraints	164
Sugar pucker	29
Backbone	109
Glycosidic bond	26
**Structure statistics**	
Violations	
Max. dihedral angle violation (°)	0.740
Max. distance constraint violation (Å)	0.110
Deviations from idealized geometry	
Bond lengths (Å)	0.011 ± 0.000
Bond angles (°)	2.545 ± 0.010
Average pairwise r.m.s. deviation** (Å)	
All RNA heavy	0.7
Triloop region (77 – 79)	0.7
Stem region (64 – 76, 80 – 92)	0.6

**Pairwise r.m.s. deviation was calculated among 10 refined structures.

The resulting structure of the simulated annealing protocol shows that the U76:A80 base pair is actually formed. However, the lack of an observable U76 imino resonance suggests increased exchange of the imino proton. Similarly, the U66:U90 mismatch in the final MD structures is nearly coplanar with two hydrogen bonds (N3-H3…O2 and O4…H3-N3), which however do not give observable imino ^1^H resonances suggesting a dynamic nature of the two nucleotides. Interestingly, the arrangement of the loop nucleobases GUG clusters into one of two possible energy minima in the final ensemble of structures. In one family of structures G79 exhibits a χ angle of around 100° and is nearly coplanar with G77 (Fig. 4C), while in the second family of structures G79 is flipped and exhibits a χ angle of around 45° (Fig. 4D). In both cases G79 is in *syn* conformation and its N7 atom forms a hydrogen bond with G77’s amino group. No stacking interactions between G79 and A80 could be observed in either of the energy minima. On the other hand, all structures exhibit efficient stacking of G77 on U76 and U78 on G77. Analysis of 100 molecular dynamics simulations reveals nearly equivalent relative populations of the two loop structures (Fig. 4C and D).

### MD simulation supports NMR results

A 400 ns MD simulation was performed with model 1 of the refined structures as a starting conformation. It has been divided into two equal parts which were analysed separately to validate the convergence of the simulation. Since both parts provided similar qualitative data, only results from the second part (200–400 ns) are shown here. The ensemble averages $\langle NO{E}_{i}\rangle$ of the simulation were calculated as $\langle NO{E}_{i}\rangle =\langle 1/{r}_{i}^{6}\rangle$. For 7 NOEs, the ensemble average was not satisfied, meaning that the average distance between the corresponding hydrogen atoms was too large. 5 out of these 7 NOEs coincide with the ones that were not satisfied in the NMR-refined structures, showing an accumulation of violations in the triloop and its direct vicinity.

The snapshots in the simulation could be reweighted such that the weighted ensemble averages satisfied all NOEs. From the 20 highest weighted structures, a set of 5 structures was chosen, in which every NOE is satisfied in at least one structure. This set was selected by i. taking the highest weighted structure and ii. including from the next highest structures the ones which reduce the number of unsatisfied NOEs. This set of structures can be found in the Supplementary Material.

The amount in which imino protons are participating in hydrogen bonds has been calculated for each donor as a fraction of the reweighted ensemble (Table 2). In agreement with the HSQC spectra acquired at 25 °C, the imino protons in the stem region from G70-G74 are entirely bound in hydrogen bonds (stability 0.98–0.99). All pairs corresponding to the spectra acquired at 0 °C had lower stabilities in the simulation. For the G68:U88 pair, which showed a weak signal in the spectrum, the hydrogen bond involving G68 as a donor had a stability of 0.90, while the one with U88 as a donor had a stability of 0.76. For the U66:U90 pair with MD values of 0.68 (U66 as donor) and 0.41 (U90 as donor) no NMR signal could be observed. The hydrogen bonds of the adjacent Watson-Crick pairs (donors U69, G89, G91 and G92) have stabilities between 0.80 and 0.95, with higher values for the pairs further away from the U:U pair, which is also in agreement with the experimental data. For the base pair adjacent to the triloop, for which no NMR signal was observed, the hydrogen bond U76:A80 was formed with a stability of 0.82. In this A:U base pair the accessibility to solvent seems the prevailing factor responsible for the absence of signals in the HSQC spectrum.

**Table 2:** Hydrogen bond stability, given for each donor/acceptor pair. Stability can have values between 0 and 1.

Donor	Acceptor	hydrogen bond stability
U66	U90 O2, O4	0.68
G68	U88 O2, O4	0.90
U69	A87 N1	0.92
G70	C86 N3	0.98
C71	C85 N3	0.99
U72	A84 N1	0.98
G73	C83 N3	0.99
G74	C82 N3	0.99
U75	A81 N1	0.87
U76	A80 N1	0.82
G77	G79 O6	0.00
G79	G77 O6	0.00
U88	G68 O6	0.76
G89	C67 N3	0.83
U90	U66 O2, O4	0.41
G91	C65 N3	0.80
G92	C64 N3	0.95

The average annotations for pairs of bases were calculated from the reweighted ensemble and are shown in Supplementary Material Figure S5. They agree qualitatively with the analysis of the imino protons. The base pairs next to the triloop, starting from pair 76U-80A, show stable interactions on their respective Watson-Crick edge. However, the two base pairs immediately next to the loop have a significant fraction of states that do not match a canonical Watson-Crick pair annotation (labelled as WW in Figure S5). A similar effect is observed for the base pairs next to the U66:U90 mismatch. The upward stackings between neighbouring bases starting from G73-G74 until G77-U78 are very stable (0.9–1.0). Stacking interactions between bases U78-G79 (downward, 0.6) and G79-A80 (outward, 0.3) are significantly less stable, indicating that the loop is highly dynamic. This is in qualitative agreement with the fact that base G79 is arranged differently in the different models obtained from annealing. The next upward stackings from A80-A81 to A87-U88 are again very stable (0.8 to 1.0).

### PDB mining for similar structures

The entire PDB was searched for fragments of NMR models in solution with some degree of structural similarity to a template built using the apical 13 nucleotides (U72-A84) of model 1. All the structures with an εRMSD <1.0 are reported in Table 3. The closest match is 2N4L, an intronic splicing silencer with a AGUGA pentaloop and 46% sequence identity with the template. One structure out of the 8 in the list is from a tRNA anticodon stem loop, although the similarity to the template should be probably ascribed to the presence of a loop with an odd number of bases. It is noteworthy that the PDB does not contain any solution structure of a tRNAHis that would have the same sequence in the triloop (GUG).

**Table 3:** εRMSD comparison of solution-state NMR structures from the PDB to 13-residue loop from MODEL 1 of inverted SINEB2/38 (U72-A84) obtained from simulated annealing in this work.

pdbID	eRMSD	Sequence	Description	residues
5LSN	0.00	UGGUUGUGAACCA	Inverted SINEB2 MODEL 1	72–84
2N4L	0.76	CUAUAGUGAAUAG	HIV-1 Intron Splicing Silencer	22–34
2KPC	0.82	AGCACAGUUUGCU	3′-untranslated region of Flaviviridae	03–15
2LBJ	0.92	GCCUUGCCAAGGU	Glycyl-tRNA anticodon stem-loop from Bacillus subtilis	03–15
2M57	0.92	GCGGUAGUUCCGC	Azotobacter vinelandii Intron 5	11–23
1A51	0.95	UGGGGUCUCCCCA	Loop E-loop D region of Escherichia coli 5 S rRNA	82–94
2MFD	0.96	GUUCGCUUAGAAC	Pentaloop from Bovine Enterovirus Vir404/03	58–70
2LPA	0.96	AGGACAUAGUCUU	Mutant of the sub-genomic promoter from Brome Mosaic Virus	02–14

## Discussion

The mouse genome contains approximately 350,000 SINE B2 sequences, dispersed throughout the genome, as independent transcriptional units or embedded in longer RNA polymerase II transcripts^33^. As independently transcribed RNAs, SINEs have been shown to possess important biological functions. SINEB2 and Alu suppress mRNA transcription upon heat shock by direct interaction with RNA polymerase II^34–36^. BC1 and SINE elements are involved in RNA localization and in the regulation of translation in neurons^37^. More recently, “exonized” or “embedded” SINE elements have been hypothesized to act as portable domains in lncRNAs, thus contributing to their biological functions^24,31,38^. A major issue to the “embedded domain” hypothesis is represented by the poor sequence conservation of lncRNAs during evolution. It is becoming evident that the sequences with higher inter-species homology do not necessary represent the functional domains of lncRNAs. This has been seen in several examples including Xist, a lncRNA involved in X chromosome inactivation^39,40^. In other cases, as in HOTAIR, the mouse and human genes share very little sequence conservation and display a completely different exon/intron organization. However, in the two species, the pattern of expression and the biological function are fully conserved^41^. Overall these data suggest that conserved lncRNAs’ functions are based on structural determinants. This concept may stand true also for embedded TEs. To address the issue of structural/functional relationship in lncRNA domains, here we take advantage of the modular organization and the well-defined biological function of AS Uchl1. This transcript contains an embedded invSINEB2 element acting as ED and crucial for its ability to increase translation of Uchl1 mRNA^28^.

We show that a large portion of the invSINEB2 secondary structure is comprised of helical structural elements. Absence of longer single stranded segments precludes any sequence-specific recognition. Therefore it is more likely that the functionality of invSINEB2 is based on its structural features. The invSINEB2 structure exhibits several internal loops and hairpins that may serve as structural motifs for specific recognition by a currently unknown partner molecule. An extended stem-loop structure, with terminal loops and internal bulges, has been previously observed for other SINE RNAs^42^, positioned either at the 5′ end, as for BC1^43^, or at the 3′ terminus, as in the case of salmon SmaI SINE^44^.

Terminal stem loop hairpin structures are often used as functional RNA motifs given their overall accessibility. This is confirmed also for the embedded invSINEB2 element of AS Uchl1. Deletion of the SL1 structural motif abolished the ability of AS Uchl1 to increase endogenous UchL1 protein levels. These results can be interpreted according to two models: 1) the SL1 terminal structural element is needed to maintain the overall structure of the invSINEB2, which in turn is necessary for AS Uchl1 activity; 2) SL1 represents *per se* a structural determinant of AS Uchl1 activity. We have found that the structure of the invSINEB2 RNA is mostly not affected by SL1 deletion, at least as predicted by mFOLD. The prediction is compatible with our chemical footprinting data that indicate a stable “basal” structure of the molecule with a more flexible region at its “apical” part. Altogether, our results support the model of the invSINEB2 as an independent folding unit acting as ED by the mean of a terminal stem loop structure. Recently, a similar model was reported for human lincRNA-p21 (hLinc-p21), a lncRNA containing an embedded SINE. This intergenic lncRNA was originally discovered in mouse as involved in stress responses mediated by p53. hLinc-p21 is a single exon gene and contains an inverted repeat Alu element (IRAlus). The authors show that structural determinants in the embedded IRAlus contribute to the nuclear localization of hLinc-p21^45^.

It should be noted that the structure/function model observed for the embedded SINEs in AS Uchl1 and hLinc-p21 seems to differ from what is observed in RNA polymerase III-transcribed SINE B2 RNA. The repression of RNA polymerase II transcription by SINE B2 RNA strictly depends on an internal single stranded region, rather than on a terminal stem loop structure^46^.

Interestingly, the impact of repeats on lncRNA functions can be also extended to repeats not derived from TEs. The Firre lncRNA contains a repeat RNA domain that is necessary and sufficient for nuclear retention^47^. It will be interesting to examine whether structural determinants contribute to the biological activity of these embedded repeats.

SINE B2 elements are not conserved in humans. We have recently identified human antisense lncRNAs that share the same domain anatomy of AS Uchl1 and function as natural SINEUPs in human cells^48^. In particular, a Free Right Alu Monomer (FRAM) embedded in AS PPP1R12A is essential for its ability to up-regulate translation. In the future, it will be interesting to assess whether embedded SINEs of human and mouse origin share the same structural determinants or, alternatively, whether different structural determinants are recruited for the same biological function.

Given the requirement of SL1 for AS Uchl1 function, we further provided a high-resolution description of its structural determinants using NMR. The SL1 hairpin features a stable G:C rich stem with a single U:U mismatch. On the other hand, its loop exhibits dynamic properties. The central GUG of the loop is followed by two A:U base pairs. According to chemical footprinting of the full length invSINEB2/183 both A:Us are accessible to methylating agents. NMR data on the truncated invSINEB2/38 clearly shows the formation of U75:A81 base pair. MD simulations also show that the U76:A80 base pair (adjacent to GUG) is stable. The discrepancies between NMR and MD results can be attributed to higher solvent accessibility of U76:A80. However, the susceptibility of the two A:U base pairs to methylation in invSINEB2/183 may suggest that SL1 is destabilized in the full length construct compared to the truncated construct used in NMR studies. The dynamic properties of the loop could be a significant factor for SINEUP activity.

SINEB2 elements are evolutionary linked to tRNA genes^49^. The secondary structure of the SL1 hairpin with a triloop followed by two unstable base pairs shares a certain degree of similarity with the anticodon arm of tRNA molecules. However, comparison of the SL1 3D structure with known structures from the PDB does not show a high degree of similarity on the high-resolution level and dismisses the possibility that tRNA mimicry could be the ground for recognition and interaction of AS Uchl1 mRNA. A similar terminal triloop structure is also seen in BC1 RNA although structure/function relationship studies have provided conflicting results on the exact contribution of structural determinants to BC1 function^50^. Analysis of transgenic mice expressing structural variants *in vivo* indicates a prominent role for the supporting basal structure in dictating BC1 biological activity^51^ suggesting a much more complex scenario than expected from the evolutionary origin of TEs.

In conclusion, we have shown that SL1 is a structural determinant required for AS Uchl1 activity. Further studies will elucidate the precise mechanism to increase protein translation by the embedded invSINEB2 and whether the SL1 motif is the sole ED portion responsible for AS Uchl1 activity. This knowledge will be important to optimize synthetic SINEUPs for their use as therapeutics of human genetic diseases such as haploinsufficiencies.

## Methods

### Plasmids

The nucleotide sequence corresponding to the invSINEB2 repeat embedded in AS Uchl1 was PCR-amplified from a pcDNA 3.1(-) plasmid expressing AS Uchl1 full length^28^. PCR fragment was digested with EcoRI and HindIII and sub-cloned into pUC19 vector for *in vitro* transcription (pUC-T7-invSINEB2). A T7 priming site was included in the forward primer. The following primers were used for cloning:

FWD EcoRI T7 invSINEB2 GAGAGAATTCTAATACGACTCACTATAGGG-CAGTGCTAGAGGAGG

REV HindIII B2 GAGAAAGCTTAAGAGACTGGAGC

AS Uchl1 mutant lacking the SL1 domain within the invSINEB2 element (Δ68–77 nt) was obtained by gene synthesis and cloned between at XbaI/HindIII sites in pcDNA 3.1(-).

### RNA synthesis and purification

183 nt RNA used for chemical footprinting was *in vitro* transcribed using a T7 RNA polymerase (Promega), rNTPs (Jena Bioscience) and pUC-T7-invSINEB2. The overnight transcription reaction was quenched with EDTA. Phenol-chloroform extraction was used to remove the proteins and extensive ultrafiltration with H_2_O through a 1000 MWCO membrane (Millipore) removed the low molecular weight components.

Similarly, 38 nt RNA construct (GGUAACCUCGUGGUGGUUGUGAACCACCAUGUGGAUGG) for NMR studies was also prepared by *in vitro* transcription, however, at a considerably larger scale and with a DNA oligonucleotide (Eurogentec) containing a T7 promoter used as a template. Last two 5′ residues of the template were 2′-OMe modified in order to keep by-products to a minimum. Unlabelled rNTPs (Jena Bioscience) and ^13^C, ^15^N-labeled rNTPs (CIL) were used for the preparation of a natural abundance and a uniformly double labelled RNA, respectively. Phenol-chloroform extraction was followed by precipitation in ethanol, the precipitate was recovered with centrifugation and by redissolving it in water. RNA was further purified with denaturing (7 M urea) PAGE electrophoresis (15%). Only bands containing the full length 38 nt RNA were excised from the gel and recovered by electroelution (Schleicher & Schuell). Finally, the RNA solution was extensively ultrafiltrated with the NMR buffer (20 mM Tris HCl buffer, 20 mM NaCl, 2 mM EDTA). The RNA concentration in the NMR samples was 0.5 mM.

The full length 183 nt invSINEB2 element and the ΔSL1 construct used for NMR were *in vitro* transcribed using a T7 RNA polymerase and corresponding pUC plasmids. After overnight transcription was quenched with EDTA the reaction mixture was loaded directly onto a GE HiPrep DEAE anion exchange column. The RNA fraction was collected and the product was precipitated in ethanol. After desalting and drying the RNA was dissolved in NMR buffer.

### Chemical footprinting

InvSINEB2 RNA was treated either with dimethyl sulfate (DMS), which methylates As and Cs, or 1-cyclohexyl-(2-morpholinoethyl)carbodiimide metho-p-toluene sulfonate (CMCT), which methylates Us and to a lesser extent Gs. 2 μL of 20% solution of DMS in ethanol were added to 20 μL of RNA solution. Modification reactions were incubated at room temperature for 2, 5, 10 or 20 min. Reactions were quenched with 475 μL of 30% β-mercaptoethanol/0.3 M NaOAc mixture. For CMCT reactions Tris HCl was added to 25 μL of RNA to adjust the pH to 8.1. 1 μL of CMCT solution (500 g/L) was added to each reaction, which were incubated at room temperature for 2, 5, 10 or 20 min. Reactions were quenched with 100 μL of 0.3 M NaOAc. DMS and CMCT solutions were prepared fresh each time minutes before treatment. The modified RNA from quenched reactions was recovered by ethanol precipitation. Dried RNA was redissolved in 20 μL H_2_O and used for primer extension. ATTO 620 5′ fluorescently labelled 18 nt DNA oligos (Eurogentec) were used as primers. The primer is complementary to the 3′ of invSINEB2 RNA and was hybridized to it by incubating the mixture at 70 °C for 5 min followed by flash cooling on ice. 80 pmol of primer and 20 pmol of RNA resulted in efficient hybridization and were used for each reaction. 200 U of Superscript II reverse transcriptase (Invitrogen) was used for primer extension. Reactions were incubated for 45 min at 42 °C following the manufacturer’s protocol and using the supplied buffer. 125 μM dNTP mixture was added to the reaction mixture. Reactions were stopped by a 20 min incubation at 70 °C. Subsequently, the modified RNA was degraded with a 10 min incubation with 2 μL (10 mg/mL) RNase A at 60 °C. The reaction mixtures were then directly loaded to large sequencing 8% 7 M urea PAGE gel.

Positive control reactions were run in a similar manner, however, with the DMS or CMCT treatment performed for half a minute at 90 °C. Negative control reactions were not treated with methylating agents. In order to position the bands within the RNA sequence classical sequencing reactions with ddNTPs were run in parallel on gels. Non-methylated RNA was used in primer extension reactions with individual ddNTPs being added to the reaction in the ratio of dNTP:ddNTP of 1:5. Bands were visualized on a Perkin Elmer ProEXPRESS imaging system.

### Cell line and transfection

Neuro2a cells were obtained from ATCC (Cat. No. ATCC-CCL-131) and maintained in culture with Minimum Essential Medium + GlutaMAX™-I (Gibco by Life Technologies, Cat. No. 41090-028) supplemented with 10% FBS (Sigma) and 1% antibiotics (penicillin/streptomycin) as suggested by the vendor. Neuro2a cells were plated in 6 well-plates the day before transfection at 80–90% confluency and transfected with AS Uchl1 plasmids using Lipofectamine® 2000 (Invitrogen™ by Life Technologies, Cat. No. 11668019) and following manufacturer’s instructions. Cells were collected at 48 hours after transfection and split in two samples for RNA extraction and Western Blot analysis.

### Western blot

For Western blot analysis, cell pellets were dissolved in Laemmli sample buffer, briefly sonicated, boiled and loaded on 12% poly-acrilamide gels. Immunoblotting was performed with the following primary antibodies: anti-UCHL1 rabbit polyclonal antibody (Millipore, Cat. No. AB1761-I,) 1:5000 and anti-β-actin (SIGMA, Cat. No. A5441), 1:2000. Signals were revealed after incubation with horseradish peroxidase-conjugated secondary antibodies (DakoCytomation, Glostrup, Denmark) in combination with Amersham^™^ ECL^™^ Detection Reagents (GE Healthcare by SIGMA, Cat. No. RPN2105). Image detection was performed with Alliance LD2-77WL system (Uvitec, Cambridge). Image quantification was done using *ImageJ* software.

### RNA isolation, Reverse Transcription and Quantitative RT-PCR (qRT-PCR)

Total RNA was extracted from cell pellets using RNeasy Mini Kit (QIAGEN) following manufacturer’s instructions. RNA was treated with on-column DNase I (QIAGEN, Cat. No. 74106) followed by a second DNase I digestion in solution (Ambion by Invitrogen, Cat. No. AM2222) to avoid plasmid DNA contamination. Single strand cDNA was prepared from 1 μg of purified RNA using iScript™ cDNA Synthesis Kit (Bio-Rad, Cat. No. 1708890) according to manufacturer’s instructions. Real Time qRT-PCR was carried out using SYBR green fluorescent dye (iQ SYBR Green Super Mix, Bio-Rad, Cat. No. 1708884) and an iCycler IQ Real time PCR System (Bio-Rad). The reactions were performed on diluted cDNA (1:20). Oligonucleotide sequences of primers used in this study for GAPDH, Uchl1 and AS Uchl1 were previously described in Carrieri *et al*.^28^. The amplified transcripts were quantified using the comparative Ct method and relative gene expression was calculated with the ΔΔCt method (Schmittgen and Livak, 2008).

### Statistical Analysis

All data are expressed as mean ± standard deviation on n ≥ 3 replicas. Statistical analysis was performed using Excel software. Statistically significant differences were assessed by Student’s *t*-test. Differences with *p* < 0.01 were considered significant.

### NMR

Spectra were recorded with the RNA either in a 100% ^2^H_2_O or 5% ^2^H_2_O, 95% H_2_O. 1D ^1^H and 2D NOESY, DQF-COSY and TOCSY NMR spectra were recorded with the natural abundance sample. 2D ^13^C and ^15^N HSQC and HCN spectra were recorded with the isotopically enriched sample. 3D NOESY-HSQC and HCCH-TOCSY spectra were also acquired, but gave poor S/N ratio due to low RNA concentration. Cross-peak intensities were therefore evaluated from 2D NOESY spectra.

All spectra were acquired on an Agilent VNMRS 600 MHz spectrometer equipped with a cryogenic probe. DPFGSE water suppression scheme was used for suppression of the water signal. All experiments were performed at temperatures of 0, 25 or 37 °C. NMR spectra were processed and analysed using VNMRJ (Agilent), NMRpipe and Sparky software (UCSF).

### Simulated annealing

NOE distance restraints for non-exchangeable protons were obtained from 2D NOESY spectra recorded at 25 °C in 100% ^2^H_2_O with mixing times ranging from 80 to 250 ms. NOE distance restraints for exchangeable protons were obtained from 2D NOESY spectra recorded at 0 °C in 5% ^2^H_2_O, 95% H_2_O with a mixing time of 250 ms. Cross-peaks were classified as strong (1.8–3.6 Å), medium (2.6–5.0 Å) and weak (3.5–6.5 Å).

Backbone torsion angle restraints (α, β, γ, ε and ζ) were used only for the helical region of the hairpin. Torsion angle restraints for α and ζ were set to 0 ± 120° to exclude *trans* conformations. Torsion angles β were restrained to 180 ± 40° (*trans*). Torsion angles ε were restrained to 235 ± 65° to exclude the unfavourable *gauche*^+^ conformation. Torsion angles γ were restrained to 60 ± 40° (*gauche*^+^). Due to the absence of H1′/H2′ cross-peaks in 2D TOCSY spectra, which indicate an N-type sugar pucker, torsion angles δ of all nucleotides were restrained to 85 ± 30°. Torsion angles χ were restrained to −120 ± 90° (*anti*) based on the intensity of intraresidual H6/H8-H1′ NOESY cross-peaks, excluding loop nucleotides.

Structure calculations were performed using AMBER 14 software with the *ff14SB* force field^52^. Initial fully extended structure was created using the leap module of Amber. The structure was then subjected to molecular dynamics (MD) calculations using a generalized Born implicit solvation model^53^. During MD NMR restraints were gradually introduced in order to obtain a hairpin structure. This was used as a starting structure for a series of simulated annealing (SA) calculations. For each of 100 SA calculation a random starting velocity was used. For the first 200 ps the molecules were held at a constant temperature of 1000 K. The molecules were then cooled to 300 K in the next 400 ps, after which the temperature was further decreased to 0 K in the next 400 ps. The force constants were 20 kcal mol^−1^ Å^−2^ for NOE distances and 500 kcal mol^−1^ rad^−2^ for torsion angles. The SHAKE algorithm for hydrogen atoms was used with a tolerance of 0.0005 Å.

A family of 10 structures with the lowest energy was subjected to a maximum of 10000 steps of steepest descent minimization and selected for further analysis. Helical parameters were determined with 3DNA 2.0 software.

### Comparison of refined structure with structures from PDB

The 13 residue hairpin (72–84) of model 1 of the refined structures was compared to solution-state NMR structures in the PDB (as of February 10th, 2016). baRNAba^54^ was used to search similar motifs, independent of sequence. Similarity was measured using the εRMSD, which reports on the relative distance and orientation of all pairs of interacting bases.

### Molecular dynamics simulations

Model 1 from the refined structures (29 nt) has been used as a starting structure for unrestrained molecular dynamics simulations (MD) of 400 nanoseconds length. The structure was solvated with water and neutralized with sodium ions. Water molecules were then replaced with ions to obtain a 0.1 mol/L concentration of NaCl, resulting in a solution of 18822 water molecules, 64 sodium and 36 chloride ions. After energy minimization, the system was subjected to a 400 ns unrestrained MD at constant temperature (T = 300 K) and pressure (P = 1 bar)^55,56^. All bonds were constrained, and snapshots were saved for later analysis every 4 ps^57^.

Simulations were done with GROMACS 4.6.7 using the AMBER force field including the latest corrections for RNA^58–60^ and ions^61^.

### Reweighting

The snapshots from the simulations were reweighted according to the maximum entropy principle^62^ such that the averaged reweighted ensemble satisfies all NOE distance restraints. The NOE intensities were classified as weak, medium and strong, corresponding to average distances r_weak_ ≤ 6.5 Å, r_medium_ ≤ 5.0 Å and r_weak_ ≤ 3.6 Å. Since NOEs are proportional to 1/r^6^ the NOEs from the simulation were calculated as  NOE_i_(t) = 1/r_i_(t)^6^ for all detectable pairs of hydrogens i, and corrected if their average was $\langle NO{E}_{i}\rangle \le 1/{r}_{i,{intensity}}^{6}=NO{E}_{i,\exp }$ (intensity = weak, medium or strong).

The weighted average value of each NOE is calculated as $$\langle NO{E}_{i}\rangle =\sum _{t}W(t)\cdot NO{E}_{i}(t)$$ with $$W(t)=\exp $$$$[-\sum _{i=1}^{N}{\lambda }_{i}\cdot NO{E}_{i}(t)]/\sum _{t\text{'}}\exp [-\sum _{i=1}^{N}{\lambda }_{i}\cdot NO{E}_{i}(t^{\prime} )]$$. Lagrangian multipliers *λ*_*i*_ were initialized to zero and updated according to $${\lambda }_{i}+=K\cdot [\langle NO{E}_{i}\rangle -(NO{E}_{i,\exp }+{\lambda }_{i}{\sigma }^{2})]$$ with the operator += indicating that *λ*_*i*_ is incremented by the amount on the right side. *K* is a constant with the value 10^−5^. σ accounts for errors in the experimental values and is equal to 0.5^63^. If *λ*_*i*_ becomes positive, it is set to zero. This cycle is repeated until all $$\langle NO{E}_{i}\rangle \ge NO{E}_{i,\exp }$$.

### Imino hydrogen bonds

Each simulation snapshot was weighted by the final set of weights W(t) which satisfied all NOE constraints. Hydrogen bonds involving imino protons (G:H1 and U:H3) were calculated as the sum of the weights of the snapshots in which the following criteria were satisfied: the distance between donor and acceptor had to be less than 3.5 Å and the angle hydrogen-donor-acceptor less than 30°. Each hydrogen bond has a stability value between 0 and 1.

### Annotations

For the reweighted ensemble, annotations were calculated using baRNAba^54^. The stability of an annotation for a pair of bases was calculated as the sum of the weights of the snapshots in which the annotation was present, and is defined in the range from 0 to 1.

### Data Availability

The coordinates for the family of the 10 lowest energy structures of the 29 nt RNA construct (CCUCGUGGUGGUUGUGAACCACCAUGUGG) have been deposited in the Protein Data Bank with the accession code 5LSN. Chemical shifts have been deposited in the Biological Magnetic Resonance Data Bank as the entry 34038.

## Electronic supplementary material

Supplementary information
