# Supplementary material for: Structural determinants of the SINE B2 element embedded in the long non-coding RNA activator of translation AS Uchl1
Source: Sci Rep. 2018 Feb 16;8:3189. doi: 10.1038/s41598-017-14908-6 (PMC5816658; doi:10.1038/s41598-017-14908-6)
Supplement: Supplementary file 1 — Supplementary information [file 41598_2017_14908_MOESM1_ESM.pdf]

SUPPLEMENTARY MATERIAL

Structural determinants of the SINE B2 element embedded in the long non-coding RNA activator of translation AS *Uchl1*

Peter Podbevšek[1,2,3], Francesca Fasolo[1], Carlotta Bon[1], Laura Cimatti[1], Sabine Reißer[4], Piero Carninci[5], Giovanni Bussi[4], Silvia Zucchelli[1,6], Janez Plavec[2,3,7,*] and Stefano Gustincich[1,8*]

[1] Area of Neuroscience, SISSA, Trieste, Italy.
[2] Slovenian NMR Centre, National Institute of Chemistry, Ljubljana, Slovenia.
[3] EN-FIST Centre of Excellence, Ljubljana, Slovenia.
[4] Molecular and Statistical Biophysics, SISSA, Trieste, Italy.
[5] Division of Genomic Technologies, RIKEN Center for Life Science Technologies, Yokohama, Japan.
[6] Department of Health Sciences, Università del Piemonte Orientale, Novara, Italy.
[7] Faculty of Chemistry and Chemical Technology, University of Ljubljana, Ljubljana, Slovenia.
[8] Department of Neuroscience and Brain Technologies, Italian Institute of Technology, Genova, Italy.

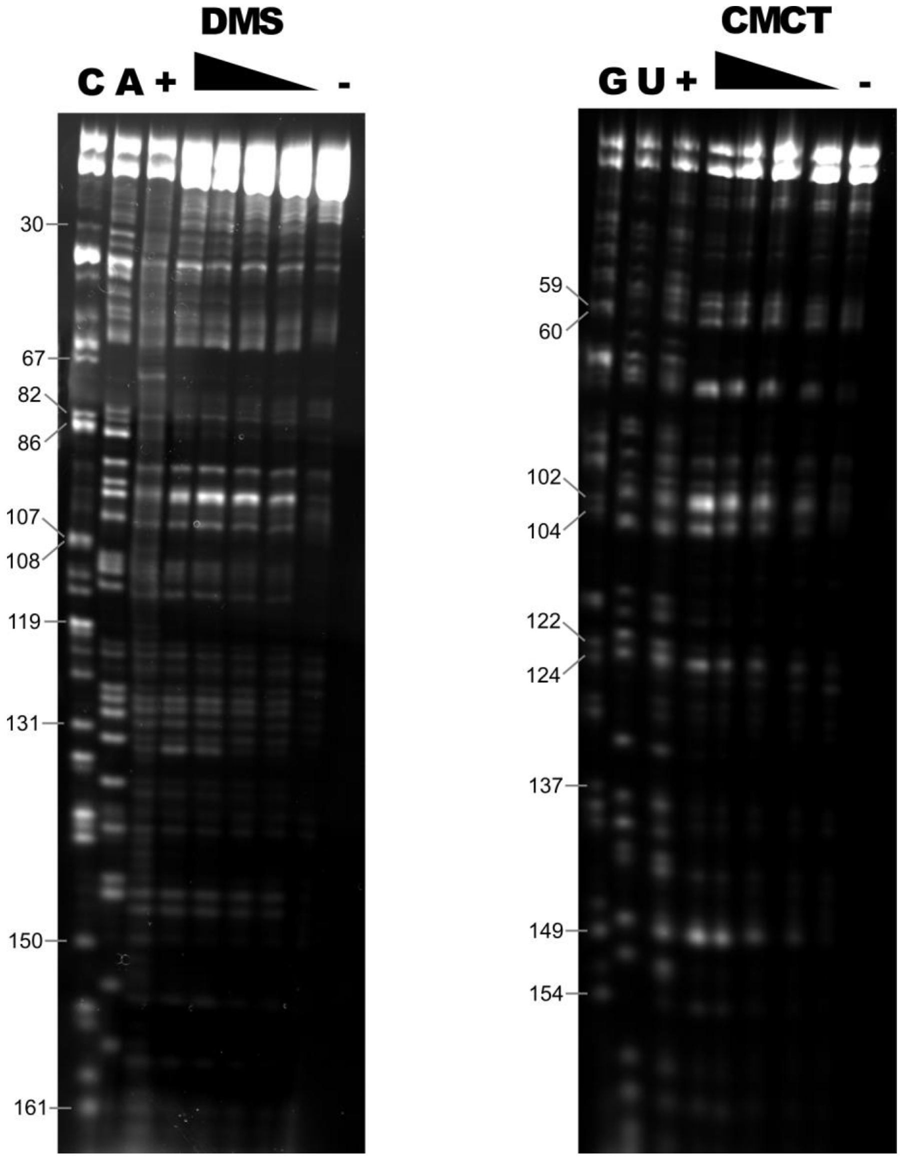

Figure S1 Chemical footprinting of the invSINEB2/183 RNA construct. (left) A fluorescent gel with C and A sequencing lanes with nucleotide numbering. DMS lanes with invSINEB2/183 RNA treated with increasing DMS concentrations with positive (+) and negative (-) controls. (right) A fluorescent gel with G and U sequencing lanes with nucleotide numbering. CMCT lanes with invSINEB2/183 RNA treated with increasing CMCT concentrations with positive (+) and negative (-) controls.

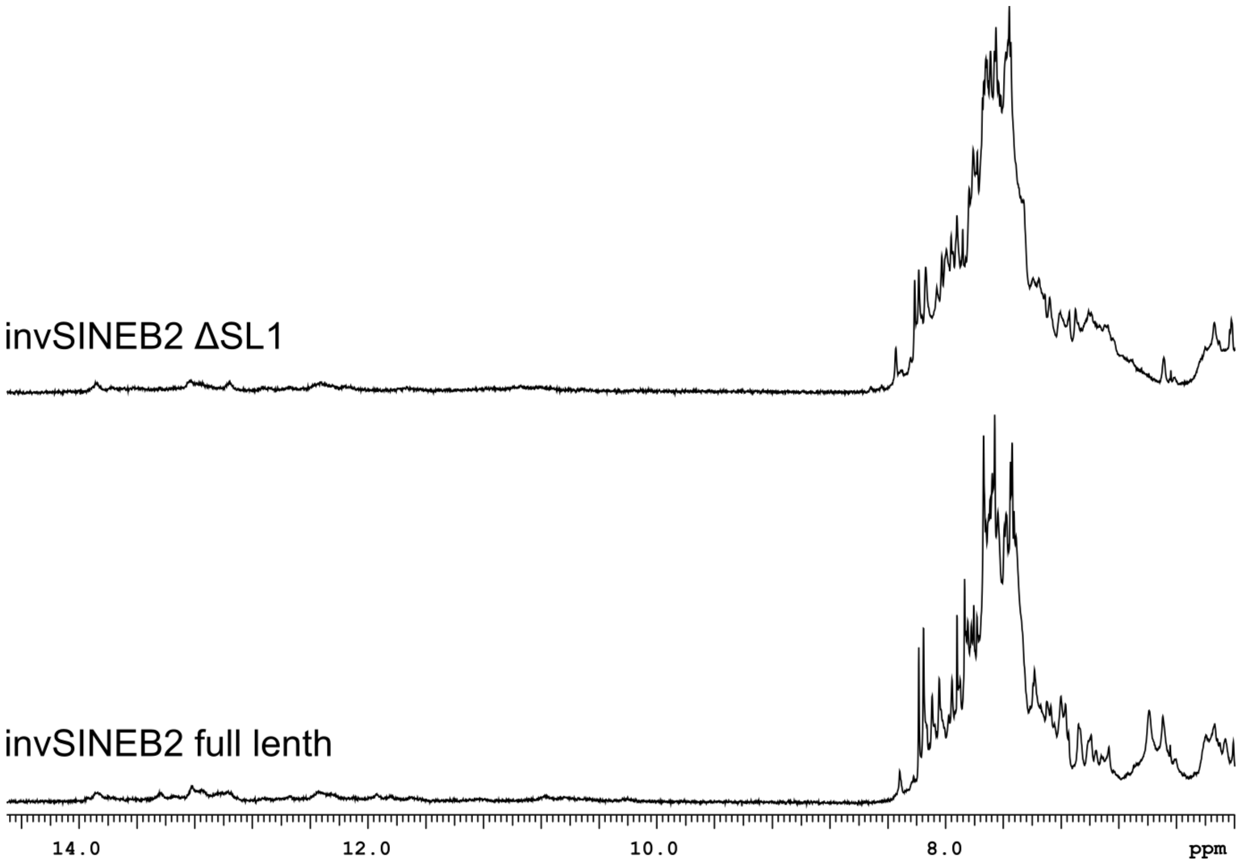

Figure S2 Imino and aromatic regions of ^{1}H NMR spectra of full length invSINEB2 RNA (183 nt) and the ΔSL1 construct (173 nt) in 5% 2H2O/95% H2O acquired at 25 °C.

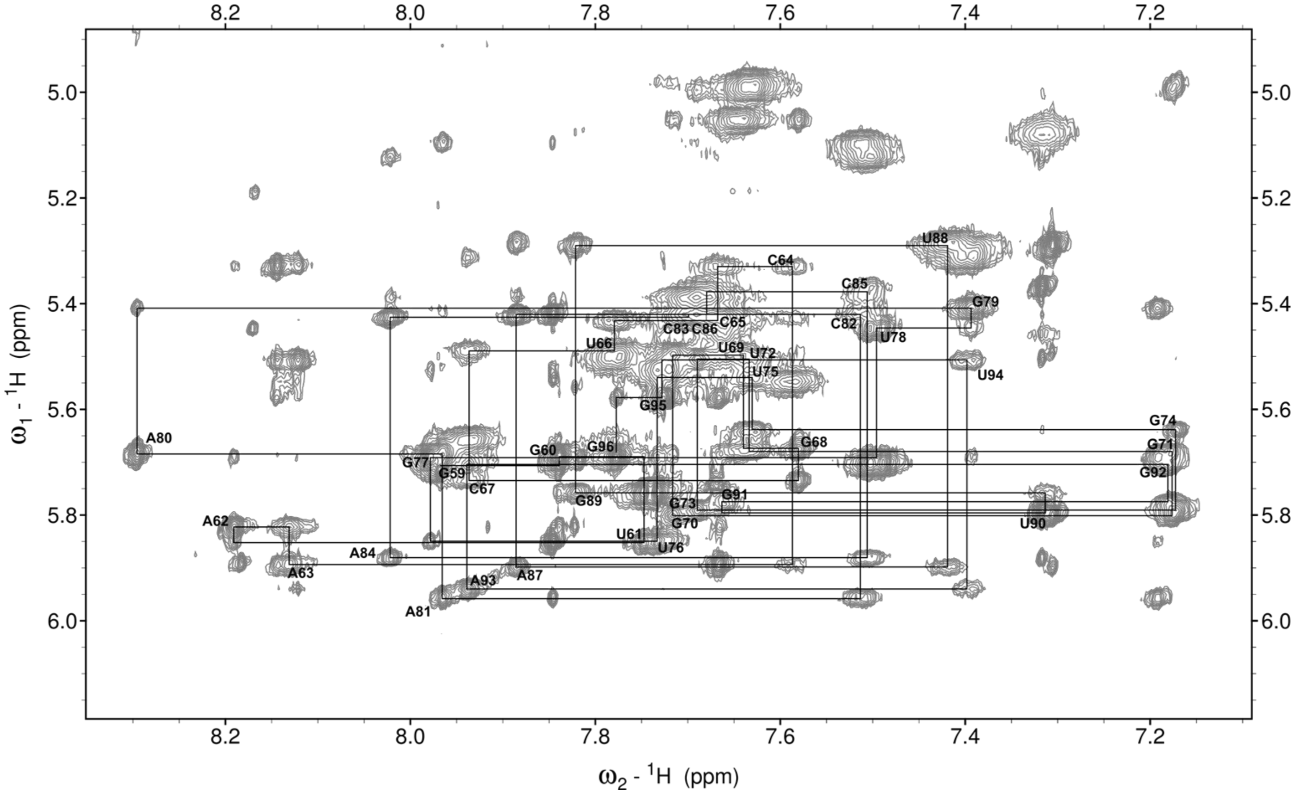

Figure S3 Assignment of relevant resonances. Aromatic-anomeric region of a 2D NOESY NMR spectrum (τ_m=250 ms) of invSINEB2/38. The sequential walk is depicted as a black line.

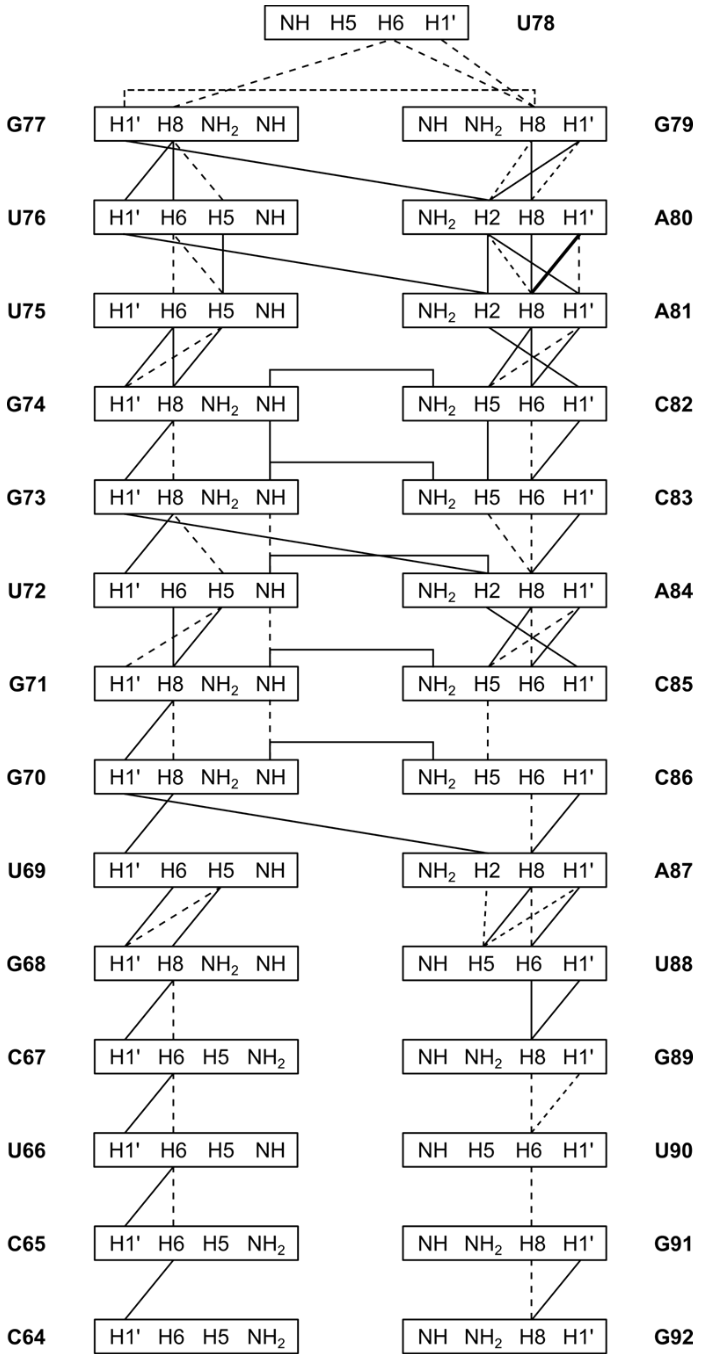

Figure S4 A diagram of strong (bold line) medium (solid line) and weak (dashed line) NOE connectivities for the invSINEB2/38 molecule.

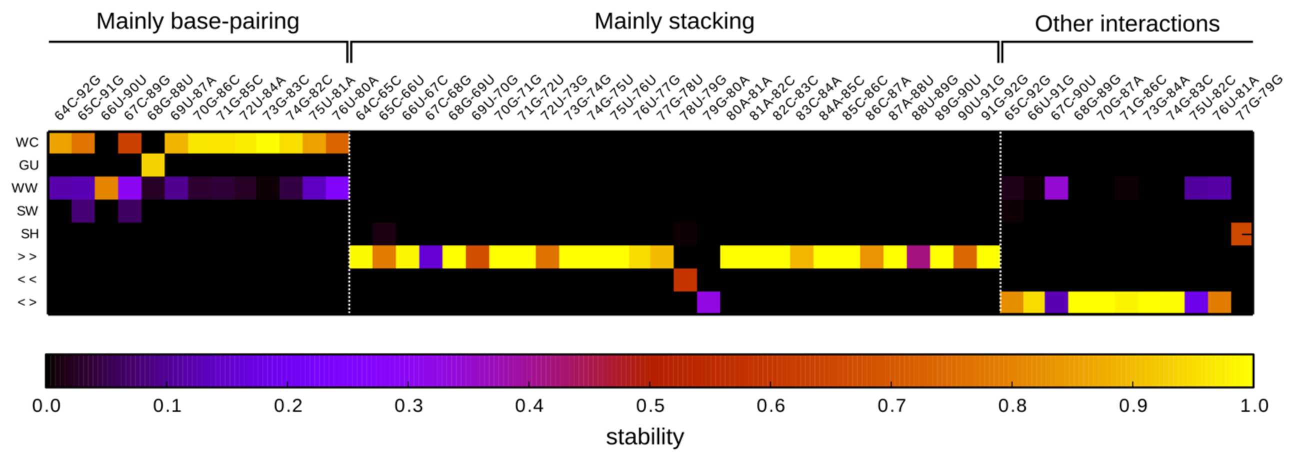

Figure S5 Stability of annotations per pair of bases, calculated with baRNAba (53) from the MD simulation. WC, GU and WW are interactions between the Watson-Crick edges of both bases. SW is a sugar-edge Watson-Crick-edge interaction, SH is a sugar-edge Hogsteen-edge interaction. >>, << and <> are stackings, in particular >> is the upward stacking found in a canonical double strand, << the downward stacking, and in <> the outward stacking.
